# Knowledge and management of temporomandibular joint disorders by general dentists in Spain

**DOI:** 10.4317/jced.55634

**Published:** 2019-08-01

**Authors:** Francisco-Javier López-Frías, Javier Gil-Flores, Victoria Bonilla-Represa, Camilo Ábalos-Labruzzi, Manuela Herrera-Martinez

**Affiliations:** 1 Associate Professor, Department of Stomatology, School of Dentistry, University of Seville, C / Avicena s / n, 41009-Seville, Spain; 2 Professor, Department of Education Science Research Methods, University of Seville, C / Pirotecnia s / n, 41013-Seville, Spain; 3 Associate Professor, Department of Stomatology, School of Dentistry, University of Seville, C / Avicena s / n, 41009-Seville, Spain; 4 PhD Contractual Professor, Department of Stomatology, School of Dentistry, University of Seville, C / Avicena s / n, 41009-Seville, Spain; 5 PhD Assistant Professor, Department of Stomatology, School of Dentistry, University of Seville, C / Avicena s / n, 41009-Seville, Spain

## Abstract

**Background:**

Given the importance of temporomandibular disorders (TMD), we tried to assess general dentists’ knowledge regarding etiology, diagnosis, and treatment in order to understand their attitude.

**Material and Methods:**

A sample of 130 general dentists answered a 16-item questionnaire on three areas – etiology, diagnosis, and management of common temporomandibular disorders – as well as a question on the need for continuous education regarding TMD management in common clinical practice in Spain. Given that the descriptive statistics achieved reflect significantly different values among means in each area, a variance analysis for repeated measurements was applied in order to contrast differences among etiology, diagnosis, and management knowledge levels.

**Results:**

The contrast test was based on Wilks’ Lambda, which assumed a value of 0.120 (F = 467.28; *p*<.001), demonstrating statistically significant differences among knowledge levels in the three dimensions. The effect size for these differences, measured by partial eta squared, was very high (η2p = 0.88). Such parameters were also analyzed to search for potential differences according to professional experience, with differences being exposed as non-significant at the 0.05 level: etiology (T = 1.60; *p* = 0.113), diagnosis (T = - 0.17; *p* = 0.868), and treatment (T = 1.10; *p* = 0.273).

**Conclusions:**

Our study found that, even though clinicians are generally skilled regarding the knowledge of the etiologic that explain the diagnosis of TMD, they have room for improvement in terms of TMD management compared to the other two areas studied. General dentists could benefit from specific educational programs enhancing TMD management skills.

** Key words:**Temporomandibular disorders (TMD), knowledge and management. Clinical competence, postgraduate, continuing professional development. Surveys, education,orofacial pain.

## Introduction

The current daily dental practice is marked by the constant evolution of knowledge and treatment options (1,2), the presence of a growing amount of patients with high expectations (3), and an increasingly higher need for cross-disciplinary cooperation (4). In order to ensure a high quality of care, educational institutions should provide dentists with the necessary skills to face daily practice challenges. Dentistry schools use multiple training strategies to turn students into dentists, including theoretical, non-clinical, and clinical education (5). In order to develop an effective dental education, education and evaluation strategies should go in line with learning outcomes based on clinical reality associated requirements (6). In support of this process, multiple skill profiles have been proposed for graduate dentists all over the world as career development instruments. In spite of social differences among regions, these profiles share the same basic skills leading to the practice of safe and independent dentistry (7). These basic skills include professionalism, communication, social skills, patient care (including evaluation, diagnosis, and treatment planning), prevention and health promotion, and scientific and clinical knowledge management. Skill profiles are aimed at providing a support plan to develop and/or compare undergraduate dentistry study plans’ learning outcomes. They are usually written by a group of renowned academic and clinical experts, and they represent an agreement among their different perspectives on dental education and practice. As a result of ongoing social change, this clinical situation is constantly changing. Therefore, experts have acknowledged the need for frequent updates of skill profiles. ‘Temporomandibular disorder’ (TMD) is a general term for musculoskeletal pain conditions associated with the joints and muscles in the masticatory system and their support tissues, including pain and dysfunction. TDMs are estimated to have a prevalence of 5-12% in the population, including adults and children. Consequently, they are regarded as an important public health issue (8). TMD treatment needs vary widely (1.5% to 30%) according to different studies (9). They are characterized by a triad of classically described clinical signs – muscle pain and/or TMJ pain, TMJ click and restriction, and mouth opening deviation. TMDs are considered as the most common orofacial pathology of non-dental origin. However, the concurrent presence of other symptoms, such as earache, headache, neuralgia, and toothache, may be related to TMDs or be present as secondary findings. Therefore, the differential diagnosis process turns TMD prevalence evaluation into a complex issue (10). TMDs come as an enigmatic entity with frequently inconsistent presentations which pose a diagnostic and therapeutic dilemma for the dentist. Even though TMD’s basic examination and clinical management are included in all Spanish dentistry schools’ syllabi, this field of orofacial diagnosis remains widely ignored in routine dental practice. Patients presenting with these disorders are often misdiagnosed, undergo various treatment rounds for non-related disorders, and are referred to other specialists without a clear idea of who they should be referred to, which often leads to frustration, lack of satisfaction, and a compromised quality of life (11,12). With our work, we try to evaluate Spanish general dentists’ awareness and attitude towards TMD so as to assess the quality of care received by patients and the need for TMD continuous education programs.

## Material and Methods

We designed a cross-sectional study with a sample of 130 general dentists working in Spain. The dentists were divided into two groups according to the number of years of professional experience they had: a group formed by dentists with 0-5 years of experience and another group composed of dentists with more than 5 years of professional practice. A questionnaire of 17 items was distributed to the dentists (13). This questionnaire contained questions related to the etiology of TMD (three), diagnosis (seven) and management (six), as well as a question about his interest in attending TMD’s continuing education programs. The same questionnaire was also delivered to 6 TMD experts, whose opinion was considered as the standard in which the examination questionnaires were evaluated, and the first sixteen questions were evaluated as correct / not correct. The last question was related to the interest they had in receiving additional education on the subject. The participants were informed about the purpose of the study and the secrecy of their answers. Only the responses of the completed questionnaires were evaluated and classified into two groups. All questionnaire data were included for analysis purposes in the SSPS 22 software (SSPS, Chicago, USA). Each element has been assigned the value 1 in case of a correct answer and 0 if it is incorrect. Descriptive statistics were used to show the general results of the frequencies, as well as the means and the standard deviation; given that in the descriptive statistics differences are reflected, statistical tests have been used that allow us to test whether these differences are significant.

## Results

This study was conducted to assess the attitude and awareness of the temporomandibular joint (TMJ) among practicing general dentists, of which 95.4% were interested in attending TMD continuing education programs. Of the questionnaires collected, only the complete ones were analyzed. 130 questionnaires were completed. These questionnaires were divided into two groups (less than 5 years of professional experience and more than 5 years) to analyze if there were differences in the 3 areas of study in both groups. The results of the answers are presented in  Table 1. The differences in knowledge in the three areas for the whole sample, N = 130, are presented in  Table 2, the data shows very different values with respect to the means in the three areas so we proceed to perform tests to contrast the means with the Wilks Lambda and the partial square eta to assess the size of the effect. Lambda de Wilks, assumed a value of 0.120 (F = 467.28, *p* <.001), demonstrating statistically significant differences between the levels of knowledge in the three areas. The effect size for these differences, measured by a partial square eta, was very high (η2p = 0.88). The T test to compare the means in the two groups, differences in knowledge according to professional experience were explored, being exposed as non-significant at the 5% level: etiology (T = 1.60. *p*= 0.113), diagnosis (T = -0.17, *p* = 0.868), and treatment (T = 1.10. *p* = 0.273). We found differences between the means in the three areas, etiology, diagnosis and levels of knowledge of the treatment. Lambda de Wilks was applied to contrast the means, and eta squared partial to evaluate the size of the effect; and Student’s T to compare knowledge among those with more and less experience ( Table 3, Table 4).

**Table 1:**
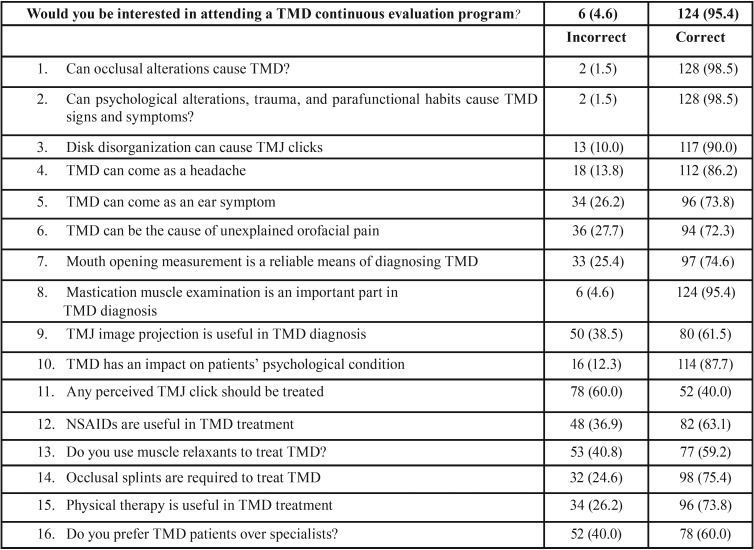
Questionnaire results and descriptive statistics.

**Table 2:**
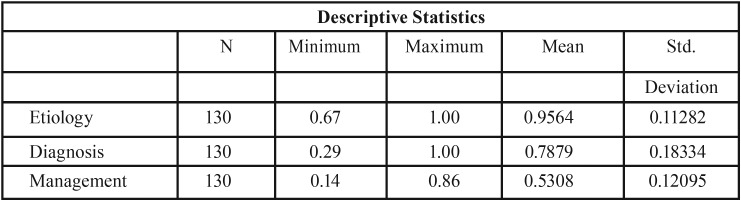
Differences knowledge in the three areas.

**Table 3:**
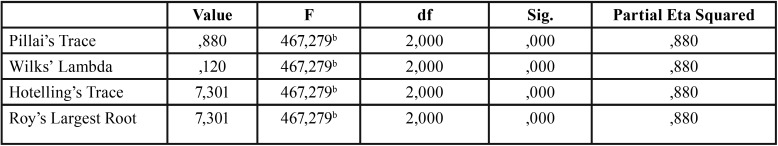
Contrast for the difference of means in the three areas.

**Table 4:**
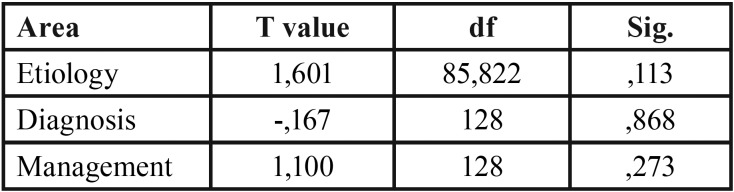
T-test for mean differences between areas based on experience.

## Discussion

98.5% of respondents believe occlusal alterations are accountable for TMD. And the fact that this number goes up to 100% among professionals with less than 5 years of professional practice proves rather significant. Similarly, 98.46% believe that parafunctional habits, trauma, and psychological factors are etiologic factors in TMD. Indeed, parafunctional habits and trauma, as well as stress, tend to spark off such processes (14). 86.25% of respondents agree with experts in that headache is associated with these disorders, without significant differences between both groups, whereas 69.23% believe earache is associated too (15). Cochrane studies (16) demonstrate that at least 33% of the population suffers from facial and temporomandibular joint pain. In a study with 60 TMD patients, Melo C. *et al.* (17) concluded that these symptoms are more severe in patients presenting with headache. Whereas 73.8% reported earache as a symptom of TMD, 26.2% did not – with a short gap between both groups (71.79% in the less than 5 years of professional experience group, and 65.38% in the more than five years group) –, as well as the presence of unexplained pain in the orofacial area (18). The disorganization of the intra-articular disk, perceived as a “click”, is considered by 90.77% of the total – 92.3% in the first group and 88.46% in the second. Moreover, whereas in a long-term, 15-year, over-190-patient study, Greene CS and Laskin DM (19) consider that this is just a sign of pre-morbidity and patients treated with conservative therapies present with pain and “click” sign improvements, other clinicians, such as Walker and Kalamchi, believe it is professionally ethical and appropriate not to treat the asymptomatic click (20). 60% of respondents would not treat any perceived “click”, the proportion increasing up to 64.1% in younger dentists. Over time, TMD symptoms (pain, psychological discomfort, physical impairment, and mandibular movement limitation) can become chronic and impact quality of life. Treatment options are limited, and they sometimes do not meet young patients’ long-term demand. Therefore, it is particularly important to identify the potential etiologic factors and their participation level early, thus allowing for the best treatment of the symptoms (21). TMD’s etiology is a multifactorial one, given the combination of psychological, physiological, structural, postural, and genetic factors, which alter the functional balance among the key components of the stomatognathic system – dental occlusion, mandibular muscles, and TMJ. Diagnosis is often based on history and physical examination. Diagnostic imaging can prove beneficial when malocclusion or intra-articular abnormalities are suspected. Mouth opening limitation is a sign considered by 73.84% of the total, and it is regarded as significant in 80.76% of professionals with over 5 years of clinical practice. In our study, muscle examination was important for 95.4% of the total. It is worth noting that only 2.56% of professionals in the first group and 7.70% in the second did not consider it as an important sign, even though this is one of the premises considered in the diagnostic criteria for temporomandibular disorders (DC/TMD) for clinical and research applications: Recommendations of the International RDC/TMD Consortium Network (22). Diagnostic imaging is only considered as important by 61.53% of respondents – 53.84% in the over 5 years of experience group. As noted by Petersson A. in the current I version of the Diagnostic Research Criteria for Temporomandibular Disorders (DRC/TMD), imaging should only be carried out when it is known that it could contribute to an appropriate diagnosis and/or a better prognosis treatment. Various techniques are used to obtain TMJ images – panoramic X-rays, simple X-rays, conventional and computed tomography (CT), digital volume tomography, cone beam computed tomography (CBCT), arthrography, and MRI (23). Psychological alterations are recognized by 87.7%, without significant differences between both groups. This goes in line with other studies (24,25) demonstrating the presence of biological elements which determine the differences in the individuals’ health. However, social and cultural differences accountable for specific aspects of health gaps should be studied and recognized as the primary receptor of patients’ health demands. Regarding TMD management, 63% of respondents used NSAID treatment, without significant differences in use between both groups. However, the use of muscle relaxants was more frequent in the group of dentists with over 5 years of professional experience than in the first group – with just 38% for the latter. The most commonly used drugs in TMD treatment are painkillers, muscle relaxants, anti-inflammatory drugs (NSAIDs), tricyclic amines, tricyclic antidepressants (TCAs), and anticonvulsants (gabapentin). Some drugs may be contraindicated in certain cases (for instance, NSAIDs) in patients with gastrointestinal disorders or NSAID-sensitive asthma. Physicians should be familiar with the potential drug interactions and side effects (short- and long-term use) of any drugs prescribed and be ready to treat adverse reactions (26,27). The relaxation splint is used by 75% of respondents, and even more in the less than 5 years of professional experience group. The occlusal stabilization splint may play an important part in long-term TMD treatment, its effect corresponding with other therapeutic modalities in the long-term follow-up. Further studies based on the appropriate use of standardized criteria and result assessment are required to better define the stabilization splint treatment modalities which can impact the persistence of its effect in the long term (28). Physiotherapy by a skilled professional, jaw exercises (for instance, relaxation, rotation, stretching, or isometric and postural exercises), superficial heat and/or cold application, massages, manual mobilization, ultrasound, low intensity laser, pulsed diathermy, and other non-invasive methods (29,30) are indicated by 73.8% of the whole sample of professionals, the proportion increasing up to 79.48% in the younger group. The most important stage in a protocol is education with cognitive awareness training and relaxation therapy, as well as self-observation by patients with masseter hypertrophy, tension headache, or bruxism. It is important to inform the patient of the cause of the disorders – especially the role played by emotional stress – and warn them about common parafunctional activities (for instance, non-functional tooth contact or oral mucosa biting). Patients should be aware of what they do with their own teeth, and when they have a bad habit, try to put an end to it (26). Mastication muscle relaxation exercises seemingly provide beneficial results as an intervention treatment for TMD in clinical practice (30). 60% of professionals referred their patient to another physician considered as more competent for the treatment of these processes. In addition, 95.4% of all professionals would be interested in attending a TMD continuous education program, with virtually no differences between both groups.

We believe in accordance with the results obtained, the fact that the management knowledge is clearly inferior to the knowledge about the other two areas according to the contrast of the media with Wilks Lambda. It is a relevant result, which should be commented on and that would lead to clear implications of paying special attention to the contents of this area in a possible training aimed at professionals.
